# Crystal structure and Hirshfeld surface analysis of *N*,*N*′-bis­(3-*tert*-butyl-2-hy­droxy-5-methyl­benzyl­idene)ethane-1,2-di­amine

**DOI:** 10.1107/S2056989018009726

**Published:** 2018-07-24

**Authors:** Pinar Sen, Sevgi Kansiz, Irina A. Golenya, Necmi Dege

**Affiliations:** a Uskudar University, Faculty of Engineering and Natural Sciences, Department of Forensic Science, 34662, Istanbul, Turkey; b Ondokuz Mayıs University, Faculty of Arts and Sciences, Department of Physics, 55139, Kurupelit, Samsun, Turkey; c Taras Shevchenko National University of Kyiv, Department of Chemistry, 64, Vladimirska Str., Kiev 01601, Ukraine

**Keywords:** crystal structure, Schiff base, hydrogen bonding, Hirshfeld surface

## Abstract

The title compound, C_26_H_36_N_2_O_2_, crystallizes in the phenol–imine form. In the mol­ecule, there are intra­molecular O—H⋯N hydrogen bonds forming *S*(6) ring motifs, and the two aromatic rings are inclined to each other by 37.9 (7)°. In the crystal, mol­ecules are linked by pairs of weak C—H⋯O hydrogen bonds, forming inversion dimers. Hirshfeld surface analysis and two-dimensional fingerprint plots indicate that the most important contributions to the crystal packing are from H⋯H (77.5%), H⋯C/C⋯H (16%), H⋯O/O⋯H (3.1%) and H⋯N/N⋯H (1.7%) inter­actions.

## Abstract 2

The title mol­ecule adopts the phenol–imine form. Two intra­molecular O—H⋯N hydrogen bonds each generate an *S*(6) ring motif. In the crystal, weak C—H⋯O hydrogen bonds link the mol­ecules into inversion dimers.

## Chemical context   

The key Schiff base condensation reaction involves simply the reaction of an amine with aldehyde to give an imine and is named after Hugo Schiff who first reported this type of reaction (Schiff, 1864 ▸). Schiff bases are considered to be an important class of organic compounds being versatile tools and having wide applications in analytical chemistry, in medicine and in biological processes, displaying anti­fungal, anti­bacterial and anti­cancer activities (Przybylski *et al.*, 2009 ▸). Schiff base ligands have also played an important role in the development of coordination and supra­molecular chemistry (Moroz *et al.*, 2012 ▸), having a chelating structure to coordinate metal ions through the imine nitro­gen and another group to form complexes (Cozzi *et al.*, 2004 ▸; Moroz *et al.*, 2008 ▸, 2010 ▸). The complexes of Schiff bases have a wide range of utilization in various areas of science such as in pharmaceutical, agriculture and industrial chemistry (Anis *et al.*, 2013 ▸).
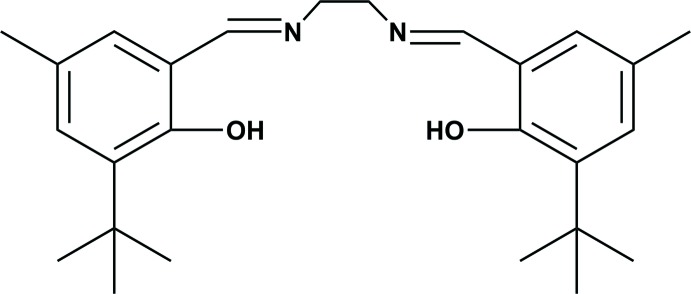


In this study, we designed a new type of Schiff base by the reaction of an aromatic aldehyde derivative and ethyl­enedi­amine to give *N*,*N*′-bis­(3-*tert*-butyl-2-hy­droxy-5-methyl­benz­yl­idene)ethane-1,2-di­amine and have also performed the synthesis, characterization and the crystal structure analysis of the target compound.

## Structural commentary   

**Figure 1:**
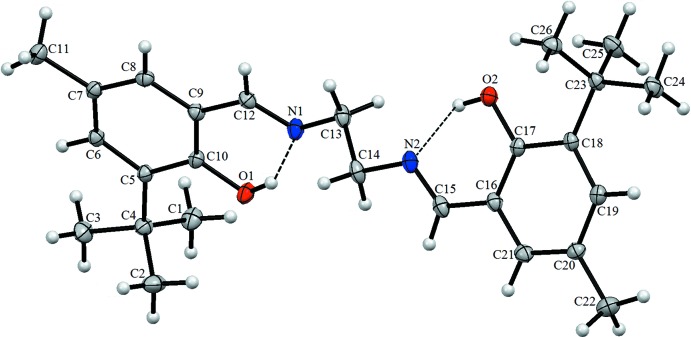
The mol­ecular structure of the title compound, showing the atom labelling. Displacement ellipsoids are drawn at the 20% probability level. Hydrogen bonds (Table 1 ▸) are shown as dashed lines.

**Figure 2:**
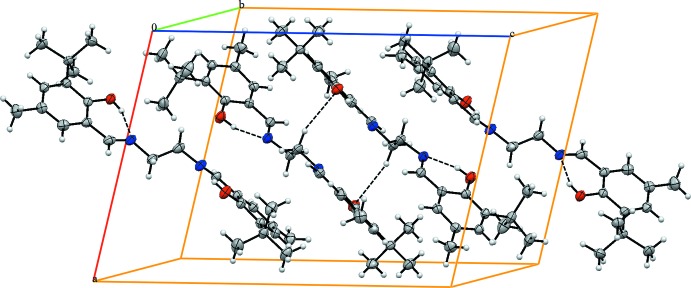
A partial view of the crystal packing. Dashed lines denote the intra­molecular O—H⋯N and inter­molecular C—H⋯O hydrogen bonds (Table 1 ▸).

The asymmetric unit of the title Schiff base compound contains one independent mol­ecule (Fig. 1 ▸). The imine groups, which display C13—N1—C12—C9 and C14—N2—C15—C16 torsion angles of 175.9 (2) and −179.6 (2)°, respectively, contribute to the general non-planarity of the mol­ecule. The aromatic ring C5–C10 is inclined to the ring C16–C21 by 37.9 (7)°. Two types of intra­molecular hydrogen bonds are observed in Schiff bases: O—H⋯N in phenol-imine and N—H⋯O keto-amine form. The present analysis shows that the title compound exists in the phenol-imine form (Fig. 1 ▸) with intra­molecular O1—H1⋯N1 and O2—H2⋯N2 hydrogen bonds,, which generate *S*(6) ring motifs, stabilizing the mol­ecular structure (Table 1 ▸ and Fig. 2 ▸). The C10—O1 and C17—O2 bond lengths [both 1.361 (3) Å] are in agreement with single bonds and support the mol­ecule being in the phenol-imine form.

## Supra­molecular features   

In the crystal, pairs of C—H⋯O hydrogen bond connect the mol­ecules into inversion dimers (Table 1 ▸, Fig. 2 ▸).

## Hirshfeld surface analysis   

**Figure 3:**
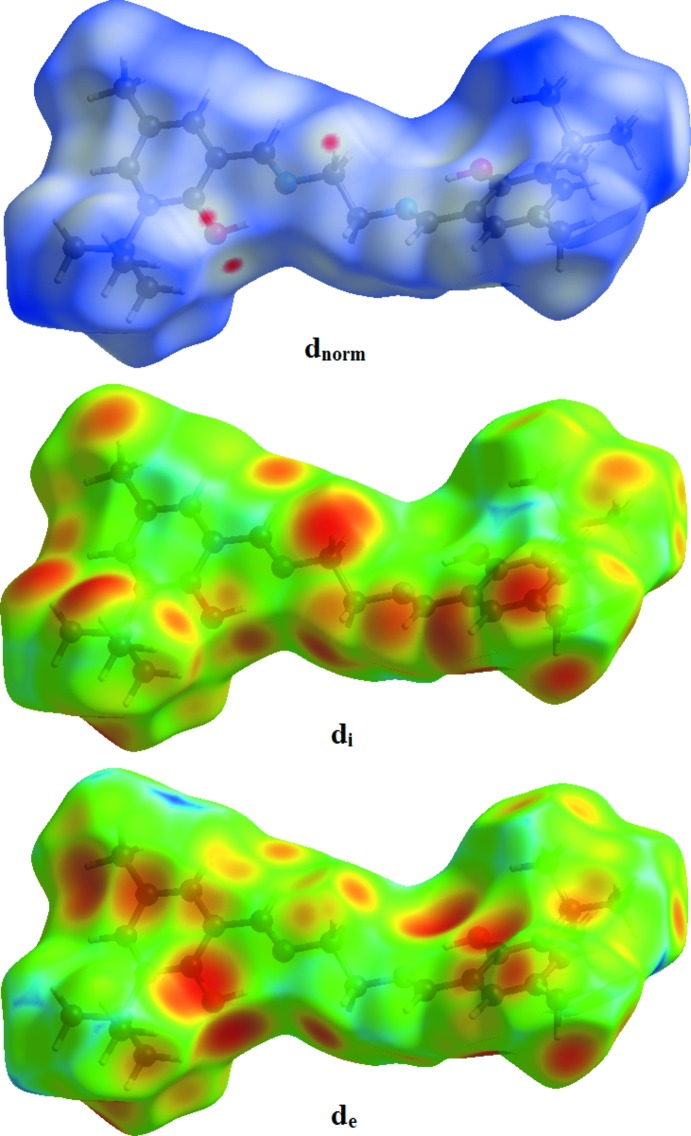
The Hirshfeld surface of the title compound mapped over *d*_norm_, *d*_i_ and *d*_e_.

**Figure 4:**
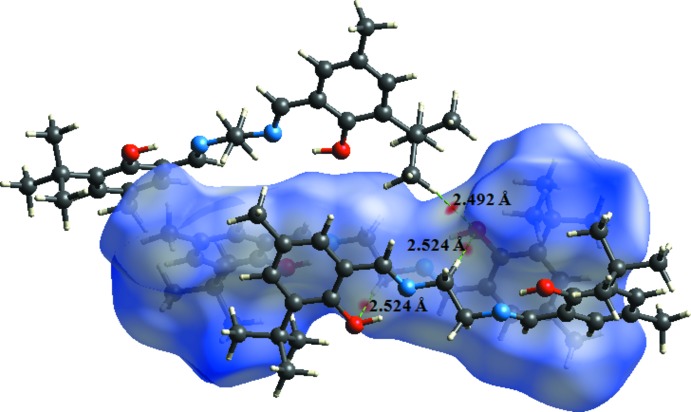
*d*_norm_ mapped on Hirshfeld surfaces for visualizing the inter­molecular inter­actions of the title compound.

Hirshfeld surface analysis was used to investigate the presence of hydrogen bonds and inter­molecular inter­actions in the crystal structure. Plots of Hirshfeld surfaces mapped over *d*_norm_, *d*_i_ and *d*_e_ using a standard (high) surface resolution with a fixed colour scale of −0.080 (red) to 1.716 (blue) a.u. are shown in Fig. 3 ▸. Red spots on these surfaces indicate strong hydrogen bonds and inter­atomic contacts (Aydemir *et al.*, 2018 ▸; Gümüş *et al.*, 2018 ▸; Hökelek *et al.*, 2018 ▸; Kansız & Dege, 2018 ▸); in the case of the title compound, these correspond to C—H⋯O hydrogen-bonding inter­actions. The red spots identified in Fig. 4 ▸ correspond to the near-type H⋯O contacts resulting from the C—H⋯O hydrogen bond.

**Figure 5:**
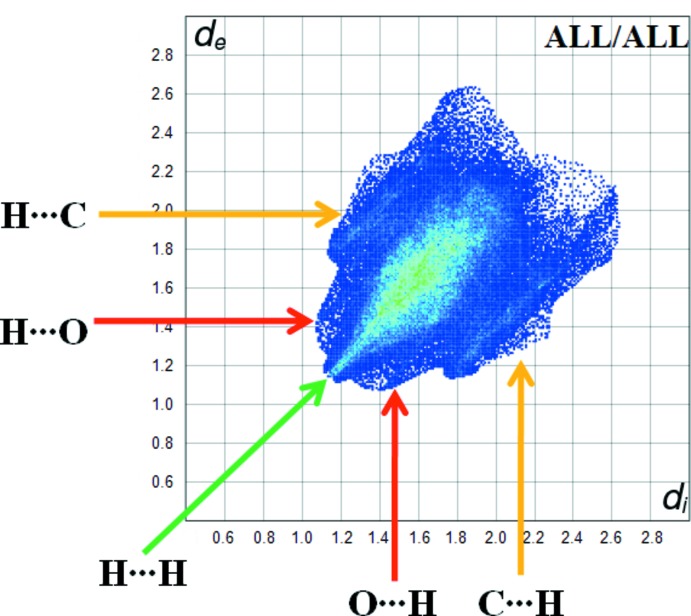
Fingerprint plot for the title compound.

**Figure 6:**
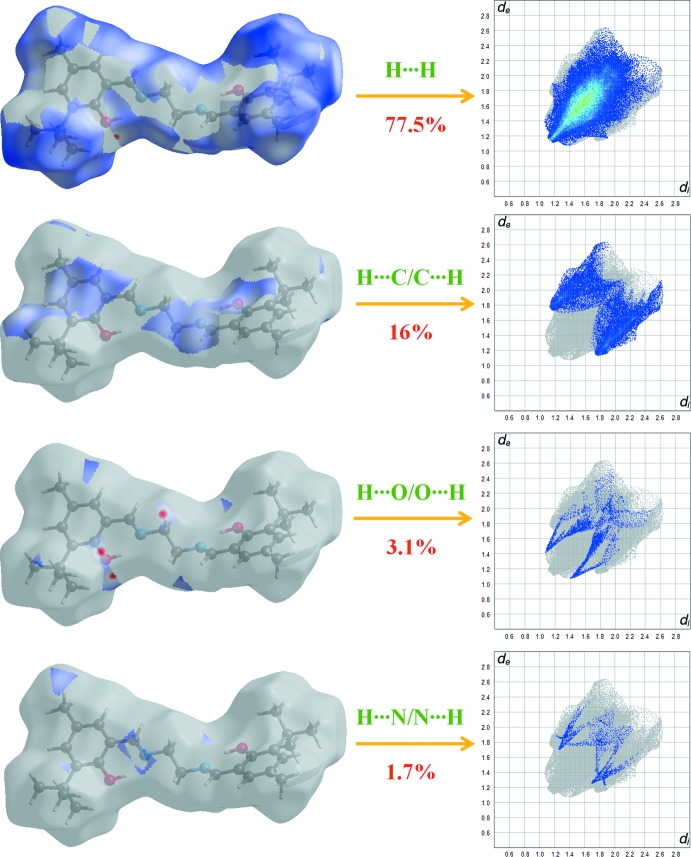
Two-dimensional fingerprint plots with a *d*_norm_ view of the H⋯H (77.5%), H⋯C/C⋯H (16%), H⋯O/O⋯H (3.1%) and H⋯N/N⋯H (1.7%) contacts in the title compound.

Fig. 5 ▸ shows the two-dimensional fingerprint [generated with CrystalExplorer (Turner *et al.*, 2017 ▸)] of the sum of the contacts contributing to the Hirshfeld surface represented in normal mode. The graph shown in Fig. 6 ▸ (H⋯H) shows the two-dimensional fingerprint of the (*d*_i_, *d*_e_) points associated with hydrogen atoms. It is characterized by an end point that points to the origin and corresponds to *d*_i_ = *d*_e_ = 1.08 Å, which indicates the presence of the H⋯H contacts in this study (77.5%). The graph shown in Fig. 6 ▸ (H⋯C/C⋯H) shows the contact between the carbon atoms inside the surface and the hydrogen atoms outside the surface of Hirshfeld and *vice* versa. The analysis of this graph shows two symmetrical wings on the left and right sides (16%). Two symmetrical points at the top, bottom left and right at *d*_e_ + *d*_i_ 2.5 Å indicate the presence of the H⋯O/O⋯H (3.1%) contacts. These are characteristic of C—H⋯O hydrogen bonds. Further, there are H⋯N/N⋯H (1.7%), C⋯C (1.2%) and C⋯N/N⋯C (0.2%) contacts.

**Figure 7:**
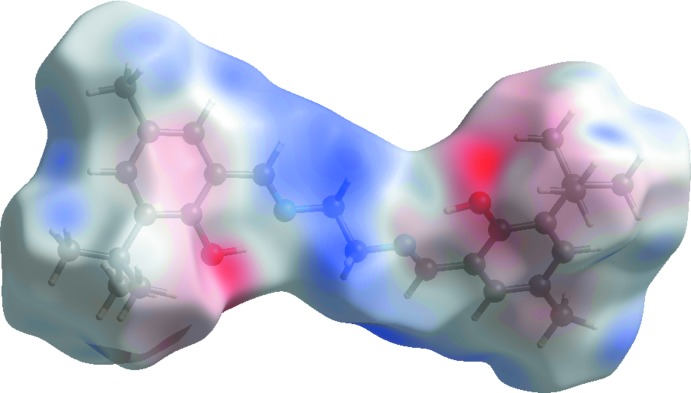
The view of the three-dimensional Hirshfeld surface of the title compound plotted over electrostatic potential energy.

A view of the three-dimensional Hirshfeld surface plotted over electrostatic potential energy in the range −0.047 to 0.041 a.u. using the STO-3G basis set at the Hartree–Fock level of theory is shown in Fig. 7 ▸; the C—H⋯O hydrogen-bond donors and acceptors are shown as blue and red areas around the atoms related with positive (hydrogen-bond donors) and negative (hydrogen-bond acceptors) electrostatic potential, respectively.

## Synthesis and crystallization   

**Figure 8:**
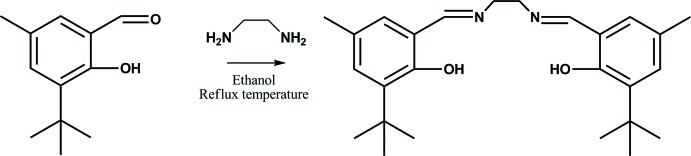
The synthesis of the title compound.

A solution of ethyl­enedi­amine (78 mg, 1.3 mmol) in methanol (30 mL) was slowly added over a solution of 3-*tert*-butyl-2-hy­droxy-5-methyl­benzaldehyde (500 mg, 2.6 mmol) in methanol (30 mL). The reaction mixture was purged with argon at room temperature and heated up to reflux temperature for 12 h. The reaction was monitored by TLC. After completion of the reaction, the mixture was cooled to room temperature. The precipitated Schiff base was filtered off and washed with diethyl ether. The resulting di­imine was recrystallized from methanol and dried under vacuum to give the desired product as a yellow powder (Fig. 8 ▸). Crystals suitable for X-ray diffraction analysis were obtained by evaporation in methanol. Yield: 85% (450 mg). FT–IR (UATR–TWO^TM^) ν max/cm^−1^: 3063 (Ar, C—H), 2957–2865 (Aliph., C—H), 1630 (C=N), 1592 (Ar, C=C), 1454–1356 (Aliph., C—C), 1265, 1206, 1029, 1043, 975, 859. ^1^H NMR (CHCl_3_) δ (ppm): 13.58 (*s*, 2H), 8.33 (*s*, 2H), 7.11 (*s*, 2H), 6.88 (*s*, 2H), 3.91 (*s*, 4H), 2.26 (*s*, 6H), 1.42 (*s*, 18H). ^13^C NMR (CHCl_3_) δ (ppm): 167.43, 158.28, 137.26, 130.79, 129.90, 126.78, 118.48, 68.19, 34.76, 29.31, 20.67. UV–Vis (CHCl_3_): λ_max_ (nm) (log ∊) 246 (3.97), 334 (3.99). MS: *m*/*z* 409.2724 [*M* + 1]^+^.

## Refinement   

Crystal data, data collection and structure refinement details are summarized in Table 2 ▸. Hydrogen atoms were positioned geometrically and refined using a riding model: O—H = 0.82 Å and C—H = 0.93–0.97 Å with *U*_iso_(H) = 1.2U_eq_(C) or 1.5U_eq_(O, C-meth­yl).

## Supplementary Material

Crystal structure: contains datablock(s) I, global. DOI: 10.1107/S2056989018009726/xu5931sup1.cif

Structure factors: contains datablock(s) I. DOI: 10.1107/S2056989018009726/xu5931Isup2.hkl

Supporting information file. DOI: 10.1107/S2056989018009726/xu5931Isup3.cml

CCDC reference: 1850298

Additional supporting information:  crystallographic information; 3D view; checkCIF report

## Figures and Tables

**Table 1:** Hydrogen-bond geometry (Å, °)

*D*—H⋯*A*	*D*—H	H⋯*A*	*D*⋯*A*	*D*—H⋯*A*
O1—H1⋯N1	0.82	1.85	2.585 (2)	149
O2—H2⋯N2	0.82	1.83	2.570 (2)	150
C14—H14*B*⋯O2^i^	0.97	2.63	3.564 (3)	162

Symmetry code: (i) 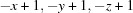.

**Table 2:** Experimental details

Crystal data	
Chemical formula	C_26_H_36_N_2_O_2_
*M*_r_	408.57
Crystal system, space group	Monoclinic, *P*2_1_/*c*
Temperature (K)	296
*a*, *b*, *c* (Å)	13.1124 (14), 9.8498 (6), 19.737 (2)
β (°)	106.892 (8)
*V* (Å^3^)	2439.1 (4)
*Z*	4
Radiation type	Mo *K*α
μ (mm^−1^)	0.07
Crystal size (mm)	0.79 × 0.45 × 0.28

Data collection	
Diffractometer	Stoe IPDS 2
Absorption correction	Integration (*X-RED32*; Stoe & Cie, 2002 ▸)
*T*_min_, *T*_max_	0.970, 0.990
No. of measured, independent and observed [*I* > 2σ(*I*)] reflections	13737, 4331, 1745
*R*_int_	0.077
(sin θ/λ)_max_ (Å^−1^)	0.596

Refinement	
*R*[*F* ^2^ > 2σ(*F* ^2^)], *wR*(*F* ^2^), *S*	0.044, 0.099, 0.78
No. of reflections	4331
No. of parameters	281
H-atom treatment	H-atom parameters constrained
Δρ_max_, Δρ_min_ (e Å^−3^)	0.10, −0.11

Computer programs: *X-AREA* and *X-RED32* (Stoe & Cie, 2002 ▸), *SHELXS97* (Sheldrick, 2008 ▸), *SHELXL2017* (Sheldrick, 2015 ▸), *ORTEP-3 for Windows* and *WinGX* (Farrugia, 2012 ▸) and *PLATON* (Spek, 2009 ▸).

## supplementary crystallographic information

### Crystal data

**Table:**

C_26_H_36_N_2_O_2_	*F*(000) = 888
*M**_r_* = 408.57	*D*_x_ = 1.113 Mg m^−^^3^
Monoclinic, *P*2_1_/*c*	Mo *K*α radiation, λ = 0.71073 Å
*a* = 13.1124 (14) Å	Cell parameters from 8399 reflections
*b* = 9.8498 (6) Å	θ = 1.6–27.6°
*c* = 19.737 (2) Å	µ = 0.07 mm^−^^1^
β = 106.892 (8)°	*T* = 296 K
*V* = 2439.1 (4) Å^3^	Prism, yellow
*Z* = 4	0.79 × 0.45 × 0.28 mm

### Data collection

**Table:**

Stoe IPDS 2 diffractometer	4331 independent reflections
Radiation source: sealed X-ray tube, 12 x 0.4 mm long-fine focus	1745 reflections with *I* > 2σ(*I*)
Detector resolution: 6.67 pixels mm^-1^	*R*_int_ = 0.077
rotation method scans	θ_max_ = 25.1°, θ_min_ = 1.6°
Absorption correction: integration (X-RED32; Stoe & Cie, 2002)	*h* = −15→15
*T*_min_ = 0.970, *T*_max_ = 0.990	*k* = −10→11
13737 measured reflections	*l* = −23→23

### Refinement

**Table:**

Refinement on *F*^2^	0 restraints
Least-squares matrix: full	Hydrogen site location: inferred from neighbouring sites
*R*[*F*^2^ > 2σ(*F*^2^)] = 0.044	H-atom parameters constrained
*wR*(*F*^2^) = 0.099	*w* = 1/[σ^2^(*F*_o_^2^) + (0.0344*P*)^2^] where *P* = (*F*_o_^2^ + 2*F*_c_^2^)/3
*S* = 0.78	(Δ/σ)_max_ < 0.001
4331 reflections	Δρ_max_ = 0.10 e Å^−^^3^
281 parameters	Δρ_min_ = −0.11 e Å^−^^3^

### Special details

**Table:**

Geometry. All esds (except the esd in the dihedral angle between two l.s. planes) are estimated using the full covariance matrix. The cell esds are taken into account individually in the estimation of esds in distances, angles and torsion angles; correlations between esds in cell parameters are only used when they are defined by crystal symmetry. An approximate (isotropic) treatment of cell esds is used for estimating esds involving l.s. planes.

### Fractional atomic coordinates and isotropic or equivalent isotropic displacement parameters (Å^2^)

**Table:**

	*x*	*y*	*z*	*U*_iso_*/*U*_eq_	
O2	0.27128 (13)	0.5225 (2)	0.43358 (8)	0.0766 (5)	
H2	0.317818	0.522865	0.471728	0.115*	
O1	0.62531 (15)	0.62851 (19)	0.83874 (8)	0.0831 (6)	
H1	0.585817	0.616104	0.798592	0.125*	
N1	0.53204 (16)	0.5008 (2)	0.72285 (9)	0.0756 (7)	
N2	0.42476 (16)	0.6117 (3)	0.53702 (10)	0.0743 (7)	
C18	0.19861 (18)	0.6627 (3)	0.33295 (11)	0.0586 (7)	
C17	0.27244 (18)	0.6428 (3)	0.39988 (11)	0.0584 (7)	
C10	0.67949 (18)	0.5121 (3)	0.86304 (11)	0.0598 (7)	
C7	0.79092 (19)	0.2731 (3)	0.91154 (12)	0.0617 (7)	
C9	0.65923 (18)	0.3954 (3)	0.82115 (11)	0.0590 (7)	
C6	0.80770 (18)	0.3918 (3)	0.95098 (11)	0.0615 (7)	
H6	0.857788	0.389270	0.995358	0.074*	
C8	0.71588 (18)	0.2780 (3)	0.84646 (12)	0.0629 (7)	
H8	0.702663	0.200207	0.818534	0.076*	
C16	0.34498 (19)	0.7427 (3)	0.43267 (11)	0.0620 (7)	
C5	0.75602 (18)	0.5135 (3)	0.92966 (11)	0.0580 (6)	
C20	0.2757 (2)	0.8891 (3)	0.33156 (13)	0.0676 (7)	
C19	0.2038 (2)	0.7877 (3)	0.30179 (12)	0.0666 (7)	
H19	0.155399	0.804515	0.257748	0.080*	
C23	0.11884 (18)	0.5534 (3)	0.29777 (11)	0.0636 (7)	
C12	0.58540 (19)	0.3967 (3)	0.75008 (12)	0.0680 (7)	
H12	0.576875	0.317491	0.723336	0.082*	
C21	0.3460 (2)	0.8645 (3)	0.39721 (13)	0.0737 (8)	
H21	0.395372	0.930893	0.418406	0.088*	
C15	0.41689 (19)	0.7228 (3)	0.50344 (13)	0.0746 (8)	
H15	0.459317	0.795248	0.525207	0.089*	
C13	0.4654 (2)	0.4944 (3)	0.64935 (11)	0.0805 (9)	
H13A	0.391091	0.506609	0.647218	0.097*	
H13B	0.472985	0.406392	0.629273	0.097*	
C4	0.7823 (2)	0.6408 (3)	0.97503 (12)	0.0711 (8)	
C14	0.49959 (19)	0.6045 (3)	0.60811 (11)	0.0832 (9)	
H14A	0.501335	0.690842	0.632017	0.100*	
H14B	0.570685	0.585398	0.605029	0.100*	
C11	0.8536 (2)	0.1455 (3)	0.93857 (13)	0.0868 (9)	
H11A	0.925020	0.155427	0.935583	0.130*	
H11B	0.820148	0.069495	0.910302	0.130*	
H11C	0.855500	0.130448	0.986974	0.130*	
C26	0.1759 (2)	0.4217 (3)	0.28836 (13)	0.0834 (9)	
H26A	0.222839	0.439553	0.260045	0.125*	
H26B	0.124098	0.354997	0.265238	0.125*	
H26C	0.216478	0.388294	0.333906	0.125*	
C24	0.0501 (2)	0.5962 (3)	0.22335 (12)	0.0914 (10)	
H24A	0.013178	0.678918	0.226697	0.137*	
H24B	−0.000727	0.526084	0.203683	0.137*	
H24C	0.095128	0.610219	0.193327	0.137*	
C1	0.6813 (2)	0.6924 (3)	0.99210 (13)	0.0961 (10)	
H1A	0.627269	0.712912	0.948806	0.144*	
H1B	0.698222	0.772806	1.020652	0.144*	
H1C	0.655724	0.623475	1.017463	0.144*	
C3	0.8677 (2)	0.6151 (3)	1.04592 (12)	0.0965 (10)	
H3A	0.842998	0.546575	1.071979	0.145*	
H3B	0.881173	0.697577	1.072939	0.145*	
H3C	0.932317	0.584927	1.037080	0.145*	
C25	0.0425 (2)	0.5255 (3)	0.34244 (12)	0.0947 (10)	
H25A	0.082538	0.494767	0.388681	0.142*	
H25B	−0.007901	0.456809	0.319794	0.142*	
H25C	0.005134	0.607358	0.346710	0.142*	
C22	0.2773 (2)	1.0227 (3)	0.29409 (14)	0.0978 (10)	
H22A	0.228066	1.018772	0.247396	0.147*	
H22B	0.347784	1.039513	0.290752	0.147*	
H22C	0.256980	1.094689	0.320326	0.147*	
C2	0.8254 (3)	0.7497 (3)	0.93535 (15)	0.1118 (12)	
H2A	0.887777	0.715967	0.924753	0.168*	
H2B	0.843630	0.829454	0.964332	0.168*	
H2C	0.771985	0.772070	0.892066	0.168*	

### Atomic displacement parameters (Å^2^)

**Table:**

	*U*^11^	*U*^22^	*U*^33^	*U*^12^	*U*^13^	*U*^23^
O2	0.0776 (13)	0.0833 (15)	0.0587 (10)	−0.0033 (11)	0.0036 (8)	0.0117 (10)
O1	0.0989 (14)	0.0721 (14)	0.0616 (10)	0.0185 (12)	−0.0031 (9)	−0.0020 (9)
N1	0.0750 (15)	0.0851 (19)	0.0555 (12)	0.0137 (14)	0.0012 (11)	−0.0056 (12)
N2	0.0655 (14)	0.095 (2)	0.0539 (13)	0.0118 (14)	0.0037 (11)	−0.0046 (12)
C18	0.0513 (14)	0.072 (2)	0.0510 (13)	0.0041 (14)	0.0131 (12)	0.0010 (13)
C17	0.0582 (15)	0.066 (2)	0.0515 (14)	0.0090 (15)	0.0163 (12)	0.0052 (14)
C10	0.0621 (15)	0.061 (2)	0.0533 (14)	0.0064 (15)	0.0125 (12)	0.0055 (13)
C7	0.0637 (16)	0.061 (2)	0.0632 (16)	0.0062 (15)	0.0224 (14)	0.0085 (14)
C9	0.0597 (15)	0.062 (2)	0.0538 (14)	0.0006 (14)	0.0146 (12)	−0.0045 (13)
C6	0.0624 (16)	0.069 (2)	0.0512 (13)	−0.0008 (15)	0.0134 (12)	0.0045 (14)
C8	0.0669 (16)	0.061 (2)	0.0648 (16)	−0.0030 (15)	0.0250 (14)	−0.0047 (13)
C16	0.0576 (15)	0.069 (2)	0.0557 (15)	0.0020 (15)	0.0117 (13)	−0.0082 (14)
C5	0.0632 (15)	0.0587 (19)	0.0502 (13)	0.0005 (15)	0.0134 (12)	0.0015 (13)
C20	0.0750 (18)	0.060 (2)	0.0699 (17)	0.0028 (16)	0.0249 (15)	−0.0021 (15)
C19	0.0644 (16)	0.074 (2)	0.0601 (15)	0.0104 (16)	0.0161 (12)	0.0016 (15)
C23	0.0519 (15)	0.081 (2)	0.0537 (13)	−0.0074 (15)	0.0080 (12)	0.0023 (13)
C12	0.0642 (16)	0.078 (2)	0.0588 (15)	−0.0074 (16)	0.0129 (13)	−0.0065 (14)
C21	0.0743 (18)	0.068 (2)	0.0782 (18)	−0.0007 (16)	0.0206 (15)	−0.0164 (16)
C15	0.0563 (16)	0.097 (3)	0.0653 (17)	0.0032 (17)	0.0097 (14)	−0.0191 (16)
C13	0.0700 (17)	0.103 (3)	0.0569 (15)	0.0106 (18)	0.0004 (13)	−0.0133 (16)
C4	0.0847 (19)	0.062 (2)	0.0578 (14)	−0.0044 (16)	0.0075 (14)	−0.0033 (14)
C14	0.0610 (16)	0.124 (3)	0.0543 (15)	0.0044 (17)	−0.0003 (13)	−0.0040 (16)
C11	0.091 (2)	0.075 (2)	0.0907 (18)	0.0168 (18)	0.0222 (15)	0.0136 (16)
C26	0.0842 (19)	0.082 (2)	0.0795 (17)	−0.0128 (18)	0.0162 (14)	−0.0094 (15)
C24	0.0814 (19)	0.110 (3)	0.0680 (16)	−0.0141 (18)	−0.0019 (14)	0.0071 (16)
C1	0.115 (2)	0.089 (3)	0.0775 (17)	0.016 (2)	0.0179 (17)	−0.0148 (16)
C3	0.109 (2)	0.096 (3)	0.0660 (16)	−0.004 (2)	−0.0047 (16)	−0.0144 (15)
C25	0.0695 (18)	0.133 (3)	0.0820 (17)	−0.0259 (19)	0.0234 (15)	−0.0024 (18)
C22	0.119 (3)	0.075 (2)	0.103 (2)	−0.005 (2)	0.0385 (18)	0.0075 (18)
C2	0.146 (3)	0.078 (3)	0.098 (2)	−0.032 (2)	0.013 (2)	0.0019 (18)

### Geometric parameters (Å, º)

**Table:**

O2—C17	1.361 (3)	C15—H15	0.9300
O2—H2	0.8200	C13—C14	1.501 (3)
O1—C10	1.361 (3)	C13—H13A	0.9700
O1—H1	0.8200	C13—H13B	0.9700
N1—C12	1.270 (3)	C4—C2	1.530 (3)
N1—C13	1.461 (3)	C4—C3	1.538 (3)
N2—C15	1.268 (3)	C4—C1	1.544 (4)
N2—C14	1.462 (3)	C14—H14A	0.9700
C18—C19	1.386 (3)	C14—H14B	0.9700
C18—C17	1.406 (3)	C11—H11A	0.9600
C18—C23	1.521 (3)	C11—H11B	0.9600
C17—C16	1.391 (3)	C11—H11C	0.9600
C10—C9	1.396 (3)	C26—H26A	0.9600
C10—C5	1.403 (3)	C26—H26B	0.9600
C7—C8	1.373 (3)	C26—H26C	0.9600
C7—C6	1.386 (3)	C24—H24A	0.9600
C7—C11	1.512 (3)	C24—H24B	0.9600
C9—C8	1.387 (3)	C24—H24C	0.9600
C9—C12	1.455 (3)	C1—H1A	0.9600
C6—C5	1.381 (3)	C1—H1B	0.9600
C6—H6	0.9300	C1—H1C	0.9600
C8—H8	0.9300	C3—H3A	0.9600
C16—C21	1.390 (3)	C3—H3B	0.9600
C16—C15	1.454 (3)	C3—H3C	0.9600
C5—C4	1.521 (3)	C25—H25A	0.9600
C20—C21	1.376 (3)	C25—H25B	0.9600
C20—C19	1.383 (3)	C25—H25C	0.9600
C20—C22	1.512 (3)	C22—H22A	0.9600
C19—H19	0.9300	C22—H22B	0.9600
C23—C26	1.535 (3)	C22—H22C	0.9600
C23—C25	1.539 (3)	C2—H2A	0.9600
C23—C24	1.542 (3)	C2—H2B	0.9600
C12—H12	0.9300	C2—H2C	0.9600
C21—H21	0.9300		
			
C17—O2—H2	109.5	C5—C4—C3	112.3 (2)
C10—O1—H1	109.5	C2—C4—C3	107.6 (2)
C12—N1—C13	118.8 (2)	C5—C4—C1	109.7 (2)
C15—N2—C14	118.3 (3)	C2—C4—C1	110.4 (2)
C19—C18—C17	115.6 (2)	C3—C4—C1	107.4 (2)
C19—C18—C23	122.8 (2)	N2—C14—C13	109.4 (2)
C17—C18—C23	121.6 (2)	N2—C14—H14A	109.8
O2—C17—C16	119.5 (2)	C13—C14—H14A	109.8
O2—C17—C18	118.6 (2)	N2—C14—H14B	109.8
C16—C17—C18	122.0 (2)	C13—C14—H14B	109.8
O1—C10—C9	119.7 (2)	H14A—C14—H14B	108.2
O1—C10—C5	118.7 (2)	C7—C11—H11A	109.5
C9—C10—C5	121.6 (2)	C7—C11—H11B	109.5
C8—C7—C6	116.7 (2)	H11A—C11—H11B	109.5
C8—C7—C11	121.8 (2)	C7—C11—H11C	109.5
C6—C7—C11	121.5 (2)	H11A—C11—H11C	109.5
C8—C9—C10	118.9 (2)	H11B—C11—H11C	109.5
C8—C9—C12	119.4 (2)	C23—C26—H26A	109.5
C10—C9—C12	121.5 (3)	C23—C26—H26B	109.5
C5—C6—C7	125.2 (2)	H26A—C26—H26B	109.5
C5—C6—H6	117.4	C23—C26—H26C	109.5
C7—C6—H6	117.4	H26A—C26—H26C	109.5
C7—C8—C9	121.9 (2)	H26B—C26—H26C	109.5
C7—C8—H8	119.0	C23—C24—H24A	109.5
C9—C8—H8	119.0	C23—C24—H24B	109.5
C21—C16—C17	118.8 (2)	H24A—C24—H24B	109.5
C21—C16—C15	120.1 (3)	C23—C24—H24C	109.5
C17—C16—C15	121.1 (3)	H24A—C24—H24C	109.5
C6—C5—C10	115.5 (2)	H24B—C24—H24C	109.5
C6—C5—C4	121.9 (2)	C4—C1—H1A	109.5
C10—C5—C4	122.5 (2)	C4—C1—H1B	109.5
C21—C20—C19	117.4 (3)	H1A—C1—H1B	109.5
C21—C20—C22	120.9 (3)	C4—C1—H1C	109.5
C19—C20—C22	121.7 (2)	H1A—C1—H1C	109.5
C20—C19—C18	124.6 (2)	H1B—C1—H1C	109.5
C20—C19—H19	117.7	C4—C3—H3A	109.5
C18—C19—H19	117.7	C4—C3—H3B	109.5
C18—C23—C26	111.0 (2)	H3A—C3—H3B	109.5
C18—C23—C25	109.9 (2)	C4—C3—H3C	109.5
C26—C23—C25	109.8 (2)	H3A—C3—H3C	109.5
C18—C23—C24	112.0 (2)	H3B—C3—H3C	109.5
C26—C23—C24	106.6 (2)	C23—C25—H25A	109.5
C25—C23—C24	107.28 (19)	C23—C25—H25B	109.5
N1—C12—C9	123.1 (3)	H25A—C25—H25B	109.5
N1—C12—H12	118.5	C23—C25—H25C	109.5
C9—C12—H12	118.5	H25A—C25—H25C	109.5
C20—C21—C16	121.6 (3)	H25B—C25—H25C	109.5
C20—C21—H21	119.2	C20—C22—H22A	109.5
C16—C21—H21	119.2	C20—C22—H22B	109.5
N2—C15—C16	123.4 (3)	H22A—C22—H22B	109.5
N2—C15—H15	118.3	C20—C22—H22C	109.5
C16—C15—H15	118.3	H22A—C22—H22C	109.5
N1—C13—C14	108.7 (2)	H22B—C22—H22C	109.5
N1—C13—H13A	110.0	C4—C2—H2A	109.5
C14—C13—H13A	110.0	C4—C2—H2B	109.5
N1—C13—H13B	110.0	H2A—C2—H2B	109.5
C14—C13—H13B	110.0	C4—C2—H2C	109.5
H13A—C13—H13B	108.3	H2A—C2—H2C	109.5
C5—C4—C2	109.3 (2)	H2B—C2—H2C	109.5
			
C19—C18—C17—O2	179.7 (2)	C23—C18—C19—C20	−179.2 (2)
C23—C18—C17—O2	−0.3 (3)	C19—C18—C23—C26	121.8 (3)
C19—C18—C17—C16	0.6 (3)	C17—C18—C23—C26	−58.2 (3)
C23—C18—C17—C16	−179.4 (2)	C19—C18—C23—C25	−116.4 (3)
O1—C10—C9—C8	179.4 (2)	C17—C18—C23—C25	63.5 (3)
C5—C10—C9—C8	0.4 (4)	C19—C18—C23—C24	2.7 (3)
O1—C10—C9—C12	3.4 (4)	C17—C18—C23—C24	−177.3 (2)
C5—C10—C9—C12	−175.6 (2)	C13—N1—C12—C9	175.9 (2)
C8—C7—C6—C5	−0.7 (4)	C8—C9—C12—N1	−178.9 (2)
C11—C7—C6—C5	178.3 (2)	C10—C9—C12—N1	−2.9 (4)
C6—C7—C8—C9	−0.1 (4)	C19—C20—C21—C16	−0.2 (4)
C11—C7—C8—C9	−179.2 (2)	C22—C20—C21—C16	179.4 (2)
C10—C9—C8—C7	0.2 (4)	C17—C16—C21—C20	1.4 (4)
C12—C9—C8—C7	176.3 (2)	C15—C16—C21—C20	−177.0 (2)
O2—C17—C16—C21	179.3 (2)	C14—N2—C15—C16	−179.6 (2)
C18—C17—C16—C21	−1.6 (4)	C21—C16—C15—N2	−175.6 (3)
O2—C17—C16—C15	−2.3 (3)	C17—C16—C15—N2	6.1 (4)
C18—C17—C16—C15	176.8 (2)	C12—N1—C13—C14	−122.3 (3)
C7—C6—C5—C10	1.3 (4)	C6—C5—C4—C2	117.3 (3)
C7—C6—C5—C4	−177.5 (2)	C10—C5—C4—C2	−61.4 (3)
O1—C10—C5—C6	179.9 (2)	C6—C5—C4—C3	−2.0 (3)
C9—C10—C5—C6	−1.1 (3)	C10—C5—C4—C3	179.3 (2)
O1—C10—C5—C4	−1.3 (4)	C6—C5—C4—C1	−121.4 (3)
C9—C10—C5—C4	177.7 (2)	C10—C5—C4—C1	59.8 (3)
C21—C20—C19—C18	−1.0 (4)	C15—N2—C14—C13	155.8 (2)
C22—C20—C19—C18	179.5 (3)	N1—C13—C14—N2	−172.3 (2)
C17—C18—C19—C20	0.8 (4)		

### Hydrogen-bond geometry (Å, º)

**Table:**

*D*—H···*A*	*D*—H	H···*A*	*D*···*A*	*D*—H···*A*
O1—H1···N1	0.82	1.85	2.585 (2)	149
O2—H2···N2	0.82	1.83	2.570 (2)	150
C14—H14*B*···O2^i^	0.97	2.63	3.564 (3)	162

Symmetry code: (i) −*x*+1, −*y*+1, −*z*+1.
